# *Glossina palpalis palpalis* populations from Equatorial Guinea belong to distinct allopatric clades

**DOI:** 10.1186/1756-3305-7-31

**Published:** 2014-01-17

**Authors:** Carlos Cordon-Obras, Jorge Cano, Jenny Knapp, Paloma Nebreda, Nicolas Ndong-Mabale, Policarpo Ricardo Ncogo-Ada, Pedro Ndongo-Asumu, Miguel Navarro, Joao Pinto, Agustin Benito, Jean-Mathieu Bart

**Affiliations:** 1 Centro Nacional de Medicina Tropical, Instituto de Salud Carlos III, Sinesio Delgado, 4, pabellón 13, Madrid 28029, Spain; 2 Instituto de Parasitologia y Biomedicina Lopez Neyra, CSIC, Avenida del Conocimiento, SN, Armilla, Granada 18100, Spain; 3 London School of Hygiene and Tropical Medicine, Keppel St, London WC1E 7HT, UK; 4 Laboratoire de Parasitologie, UMR/CNRS Chrono-environnement 6249, 19 rue Ambroise Paré, Besançon Cedex 25030, France; 5 Centro de Referencia para el Control de Endemias, Instituto de Salud Carlos III, Malabo, Equatorial Guinea; 6 Centro de Malária e outras Doenças Tropicais, Instituto de Higiene e Medicina Tropical, Universidade Nova de Lisboa, Rua da Junqueira 96, Lisbon 1349-008, Portugal

**Keywords:** *Glossina palpalis palpalis*, Luba, Equatorial Guinea, Tsetse, Trypanosomiasis, Phylogeny, Bioko

## Abstract

**Background:**

Luba is one of the four historical foci of Human African Trypanosomiasis (HAT) on Bioko Island, in Equatorial Guinea. Although no human cases have been detected since 1995, *T. b. gambiense* was recently observed in the vector *Glossina palpalis palpalis*. The existence of cryptic species within this vector taxon has been previously suggested, although no data are available regarding the evolutionary history of tsetse flies populations in Bioko.

**Methods:**

A phylogenetic analysis of 60 *G. p. palpalis* from Luba was performed sequencing three mitochondrial (COI, ND2 and 16S) and one nuclear (rDNA-ITS1) DNA markers. Phylogeny reconstruction was performed by Distance Based, Maximum Likelihood and Bayesian Inference methods.

**Results:**

The COI and ND2 mitochondrial genes were concatenated and revealed 10 closely related haplotypes with a dominant one found in 61.1% of the flies. The sequence homology of the other 9 haplotypes compared to the former ranged from 99.6 to 99.9%. Phylogenetic analysis clearly clustered all island samples with flies coming from the Western African Clade (WAC), and separated from the flies belonging to the Central Africa Clade (CAC), including samples from Mbini and Kogo, two foci of mainland Equatorial Guinea. Consistent with mitochondrial data, analysis of the microsatellite motif present in the ITS1 sequence exhibited two closely related genotypes, clearly divergent from the genotypes previously identified in Mbini and Kogo.

**Conclusions:**

We report herein that tsetse flies populations circulating in Equatorial Guinea are composed of two allopatric subspecies, one insular and the other continental. The presence of these two *G. p. palpalis* cryptic taxa in Equatorial Guinea should be taken into account to accurately manage vector control strategy, in a country where trypanosomiasis transmission is controlled but not definitively eliminated yet.

## Background

Following the London Declaration on Neglected Tropical Diseases, Human African Trypanosomiasis (HAT) has been targeted for elimination by 2020 [1]. As *Trypanosoma brucei gambiense* infection actually causes 98% of the total HAT cases (the remaining are due to *T. b. rhodesiense*), attention must be focused on this subspecies. Among the elimination strategies, vector control can play an important role [2-4], especially in isolated populations, which can be targeted for direct intervention avoiding the reinvasion from neighbouring zones. Islands represent an ideal setting for such strategies as demonstrated by the eradication of *Glossina* sp. in Unguja [5] and in Principe Islands [2] after a few years of sustained control.

Tsetse flies of the *palpalis* group (*Nemorhina* subgenus) are major vectors of *T. b. gambiense* in West Africa [2]. This group comprises two allopatric subspecies: *G. p. palpalis* and *G. p. gambiensis*, which probably derived from an ancestral *palpalis* species which was isolated in several geographic points when its riverine habitat declined during the last glacial maximum [6,7]. Cumulative evidences support the recognition of *G. p. gambiensis* and *G. p. palpalis* as valid specific taxa. For example, using data from the mitochondrial gene cytochrome oxidase 1 (COI), the average genetic distance observed between *G. p. palpalis* and *G. p. gambiensis* sequences was 6.6%, which is well above the threshold of 2% divergence for inter-species comparisons [8-11]. Moreover, experimental crosses between these subspecies yielded sterile males in the offspring [12]. The phylogenetic situation is more complex since recent genetic analyses suggested the existence of at least two distinct cryptic species within *G. p. palpalis*[10,11]. One circulates in the Western part of Africa (named as Western African Clade or WAC), and the other in the continental part of Equatorial Guinea and the Democratic Republic of Congo (DRC) (Central African Clade or CAC). According to the available data, both types are sympatric in the Fontem focus of Cameroon [11].

In Equatorial Guinea, four historical HAT foci are classically defined: one insular (Luba, on Bioko Island) and three on the mainland (Rio Campo in the north; Mbini in the centre and Kogo in the south) [13]. Due to sustained control measures, less than 10 HAT cases are being detected every year in the three continental foci since 2009 and no cases have been recorded since 1995 in Luba. Vector control activities were never implemented in Bioko Island, and parasite elimination in humans relied on active screening of the population at risk and systematic treatment [14]. Therefore, high densities of *G. p. palpalis* have been observed in some localities at the south of Luba district and moderate densities in others of the epicentre of the focus [15]. Moreover the presence of *T. b. gambiense* has also been reported in tsetse flies of Luba despite the absence of human infections, which could be attributed to the existence of reservoirs in the wild fauna, cryptic human infections and/or low sensitivity of available diagnostic tools [15-17]. Because vector control is a key parameter to completely eradicate the parasite [2,4,5] a deep knowledge of the biology of the tsetse fly is a crucial prerequisite. In such a context, the genetic characterization of the *Glossina palpalis palpalis*, never performed so far in Luba, has become indispensable.

In this study we combine information from both mitochondrial DNA (mtDNA- COI, ND2 and 16S- genes) and nuclear ribosomal (rDNA-ITS1) markers to investigate the phylogeographic origin of *G. p. palpalis* in the focus of Luba, Bioko Island, using tsetse flies samples captured in a previous epidemiological study [15]. MtDNA has been extensively used in population and evolutionary biology of insects [18-20] and metazoa in general [21] due to their particular features: relative ease isolation, simple sequence organization, maternal inheritance, absence of recombination and rapid rate of sequence divergence allowing discrimination of recently diverged lineages [22]. On the other hand, the rDNA internal transcribed spacer 1 (ITS1) is a useful marker for both closely related species and also intraspecific populations of insects [23-25].

## Methods

### Sample collection

Fly sampling was carried out in September/October 2007 from five areas known to harbour *G. p. palpalis* (Avendaño, Drumen, Fortuny, Boloco and Las Palmas). We employed monopyramidal traps [26], which have been successfully applied for vector control and entomological surveys in Equatorial Guinea [27-29]. Details about trap distribution are provided elsewhere [15]. Tsetse flies collected were individually stored in absolute ethanol in the field until processed in the laboratory. Species identification was undertaken using the key of Brunhes et al. [30]. Tsetse flies were sent to the National Centre of Tropical Medicine, Institute of Health Carlos III (Madrid, Spain) for subsequent molecular analysis.

### Molecular analysis

DNA was extracted from whole flies with SpeedTools Tissue DNA Kit (Biotools, B & M Labs, S.A) following the manufacturer instructions. We analysed three mtDNA (ND2, COI and 16S) and one nuclear (ITS1) markers in our study. COI, 16S and ITS1 sequences were amplified with primers described previously [11,31] whereas new specific primers for ND2 gene from *G. p. palpalis* were designed (Additional file 1: Table S1).

PCR reactions were performed with 2 μl of each template DNA, 1X buffer (10 mM Tris–HCl, 1.5 mM MgCl_2_, 50 mM KCl, pH 8.3), 100 μM of each dNTP, 0.5 μM of each primer, 1 U of Fast Start Taq DNA polymerase (Roche) and double distilled water (DDW) until reaching 50 μl final volume. The thermal cycling programme started with initial denaturation step of 2 minutes at 95°C, followed by 35 cycles (30 seconds at 95°C, 30 seconds at 55–60°C and 1 minute at 72°C) and a final extension step of 5 minutes at 72°C. Results were visualized in 2% agarose gel stained with ethidium bromide under UV irradiation. After this check, we sent the amplified products to Secugen Sequencing and Molecular Diagnostics (Madrid, Spain) where they were sequenced using Sanger method.

Forward and reverse strands of all sequences were manually inspected with Sequence Scanner software v1.0 (Applied Biosystems© 2005). Sequences were trimmed and aligned using ClustalW Multiple Alignment algorithm of BioEdit Sequence Alignment Editor version 7.0.9.0 [32]. All sequences obtained in this work are available in GenBank with the following accession numbers: KF597286-91 (COI), KF597292-5 (ND2), KF597296 (16S) and KF597297-8 (ITS1).

### Phylogenetic analysis

MEGA version 5 software [33] was used to calculate the pairwise and average distances expressed as number of nucleotide substitutions per site. Phylogeny reconstruction was performed with Maximum Likelihood (ML), Distance Based Neighbor-Joining (NJ) and Bayesian Inference (BI) methods. Model of evolution was inferred from data using the Find DNA/Protein Model tool of MEGA5. This software implements the Bayesian Information Criterion (BIC) [34] and corrected Akaike Information Criterion (AIC) [35] to measure the goodness-of-fit of the 24 models available. Unless otherwise specified, we applied the model selected by BIC to perform phylogenetic analysis (Table 1). For these three inference methods, all positions containing the missing data were eliminated.

**Table 1:** Summary of models chosen in phylogenetic analysis

**Marker**	**COI**			**ND2**			**COI + ND2**		
**Inference method**	**NJ**	**ML**	**BI**	**NJ**	**ML**	**BI**	**NJ**	**ML**	**BI**
**Model**	T92 + Γ		TN93 + Γ	T92 + Γ		HKY + Γ	TN93 + Γ		
**BIC**	4097,84		4101,20	2186,19		2193,06	5311,13		
**AIC**	3592,74		3572,45	2040,86		2034,53	5038,40		
**-Lnl**	1732,16		1719	998,34		993,16	2484,13		
**Length**	622			501			1123		
**Number of haplotypes**	30			9			14		
**Overall mean distance (SE)**	0.0176 (0.0062)			0.0093 (0.0064)			0.0149 (0.0053)		

NJ: distance-based Neighbor-Joining, ML: Maximum Likelihood, BI: Bayesian Inference, T92: Tamura 3-parameter model [36], TN93: Tamura-Nei model [37], HKY: Hasegawa-Kishino-Yano model [38], Γ: assuming Gamma distribution (five discrete gamma categories), BIC: Bayesian Information Criterion (score), AIC: corrected Akaike Information Criterion (score), -Lnl: Maximum Likelihood value, SE: Standard Error.

MEGA5 software was also used to construct ML and distance based trees of COI, ND2 and concatenated (COI + ND2) sequences. For ML, the bootstrap method (500 replications) was chosen to test the robustness of the trees [39]. The selected substitution model was applied and rates among sites were treated as Gamma distributed with five discrete categories. We assumed no invariant sites. The ML heuristic method employed was the Nearest-Neighbor-Interchange (NNI).

Distance trees were constructed using the NJ method [40]. Gamma shape parameter was estimated from data. The bootstrap consensus tree was inferred from 2000 replicates.

BI was implemented with BEAST software [41]. This software uses Metropolis-Hasting Markov Chain Monte Carlo algorithm [42]. The default settings were generally used (1x10^7^ generations and log parameters sampled each 1000 steps). The first 25% of trees generated was discarded as ‘burnin’. Yule process was implemented as tree prior, a simple model appropriate when studying speciation [43,44].

### Assessment of genetic diversity

Under our sampling conditions, we assume that undetected haplotypes can exist. In order to assess the number of these unseen haplotypes in our studied area (Luba focus), we used two estimators that calculate haplotype richness: i) the first-order Jackknife richness estimator [45] calculated as followed: Jack1 = S_0_ + a1(N-1)/N, where S_0_ = observed number of haplotypes, a1 = number of haplotypes detected in only one fly, N = total number of flies and ii) the Bootstrap richness estimator [46] boot = S_0_ + Σ(1-p_i_)^N^, where S_0_ = observed number of haplotypes, p_i_ = frequency of *i*th haplotype and N = total number of flies. Analysis was conducted in R software, with the specific ‘vegan’ package (http://cran.r-project.org, http://vegan.r-forge.r-project.org).

### Phylogeographic analysis

To infer the haplotype relationships within the data sets, the median-joining network algorithm available in NETWORK v4.5.1.0 was performed [47], which combines the topology of a minimum spanning tree with a parsimonious search for the missing haplotypes.

## Results

### Mitochondrial markers

**Figure 1:**
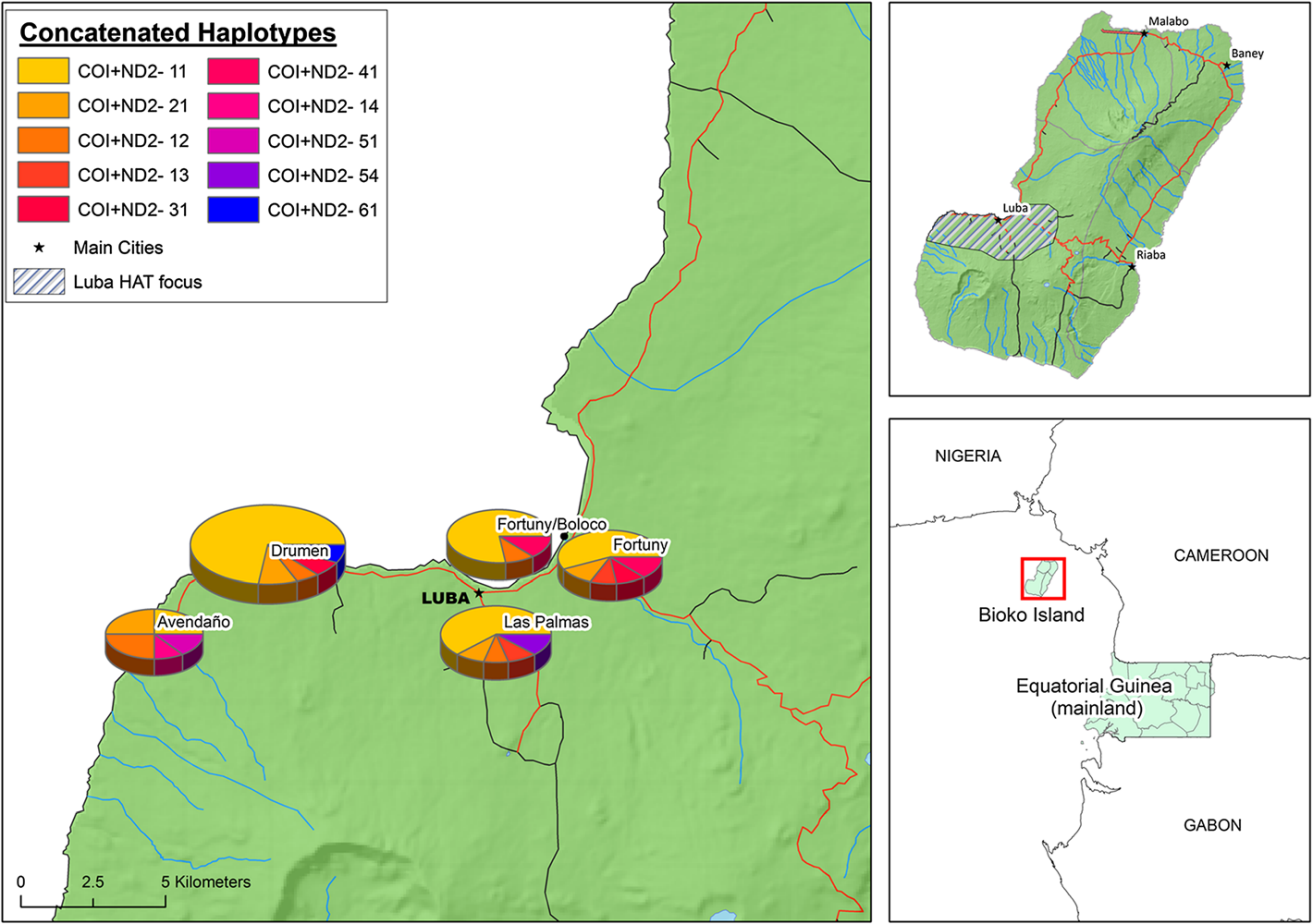
**Distribution of *G. p. palpalis *** **COI + ND2 concatenated haplotypes in Luba focus.**

We amplified the mtDNA of COI (622 bp), ND2 (501 bp) and 16S (213 bp) genes for a total of sixty tsetse flies, all coming from Equatorial Guinea. Figure 1 (COI + ND2) and Table 2 show the distribution of mtDNA haplotypes regarding the sampling location. In COI gene six different haplotypes were observed with an overall genetic distance of 0.4%, ranging from 0.2% to 0.8%, in terms of number of base substitution per site. Haplotype 1 predominated over the rest (44/60), haplotype 2 was found in 9 individuals, whereas the other haplotypes were found once or twice (Table 3). For ND2, fifty-four sequences were obtained and four haplotypes were detected with an average distance of 0.4%, ranging from 0.2% to 0.5%. Haplotype 1 was the most common (45/54). Concatenated COI + ND2 yielded 10 different haplotypes and revealed that 61.1% of individuals (33/54) shared the same pattern (haplotype 11, i.e. haplotype 1 at both COI and ND2).

**Table 2:** Distribution of the mtDNA haplotypes within Luba focus

	**COI haplotypes**						**ND2 haplotypes**				**COI + ND2 haplotypes**									
	**1**	**2**	**3**	**4**	**5**	**6**	**1**	**2**	**3**	**4**	**11**	**21**	**12**	**13**	**31**	**41**	**14**	**51**	**54**	**61**
**Las Palmas**	8	1			1		7	1	1	1	6	1	1	1					1	
**Fortuny (Boloco)**	9			1			8	1			7		1			1				
**Fortuny**	7	1	1	1			8		1		5	1		1	1	1				
**B. Drumen**	14	2	1			1	17	1			13	2	1		1					1
**B. Avendaño**	6	5			1		5	2		1	2	2	2				1	1		
**Total**	44	9	2	2	2	1	45	5	2	2	33	6	5	2	2	2	1	1	1	1

For each location is detailed the number of times a given haplotype has been obtained.

**Table 3:** Distribution of ITS1 genotypes within Luba focus

	**ITS genotypes**				
**Village**	**Genotype 1: (TA)**_**2**_**C (AT)**_**9**_		**Genotype 2: (TA)**_**10**_		**Total number of samples**
**Las Palmas**	9	90,0%	1	10,0%	10
**Fortuny/Boloco**	6	66,7%	3	33,3%	9
**Fortuny**	7	70,0%	3	30,0%	10
**B. Drumen**	14	77,8%	4	22,2%	18
**B. Avendaño**	8	66,7%	4	33,3%	12
**Total**	44	74,6%	15	25,4%	59

For each location is detailed the number of times a given genotype has been obtained.

Analysis of genetic diversity seemed to be assessed with a reasonable accuracy. The number of estimated haplotypes, n = 13.93 and 11.86 calculated with Jackknife and Bootstrap estimators respectively, was close to the number of observed haplotypes (n = 10) when all the samples are considered as a unique population (Additional file 2: Table S2). This means that the haplotype structure of the population is correctly estimated even with low sample size and underestimation of the haplotype richness.

Since very limited data are available regarding the ND2 and 16S sequences in *G. p. palpalis* from different geographic origins, we decided to focus the phylogenetic reconstruction in COI gene. To construct more comprehensive ML, distance based NJ and BI trees we included previously published sequences of *G. p. palpalis* from DRC (EU591840-2), Cameroon (EU591829-31, EU591860 and EU591865), Liberia (EU591857-9), Togo (EU591838-9), Ivory Coast (EU591846-8 and EU591832-3), Burkina Faso (EU591856) and two continental foci of Equatorial Guinea, Kogo and Mbini (EU591825 and FJ767873-6). The source of these sequences is detailed elsewhere [10,11]. *G. morsitans* (GQ255905) was used to root the tree and *G. p. gambiensis* (EU591851) sequence was also placed as outgroup. Analysis was based on a total fragment of 622 bp of the COI gene.

**Figure 2:**
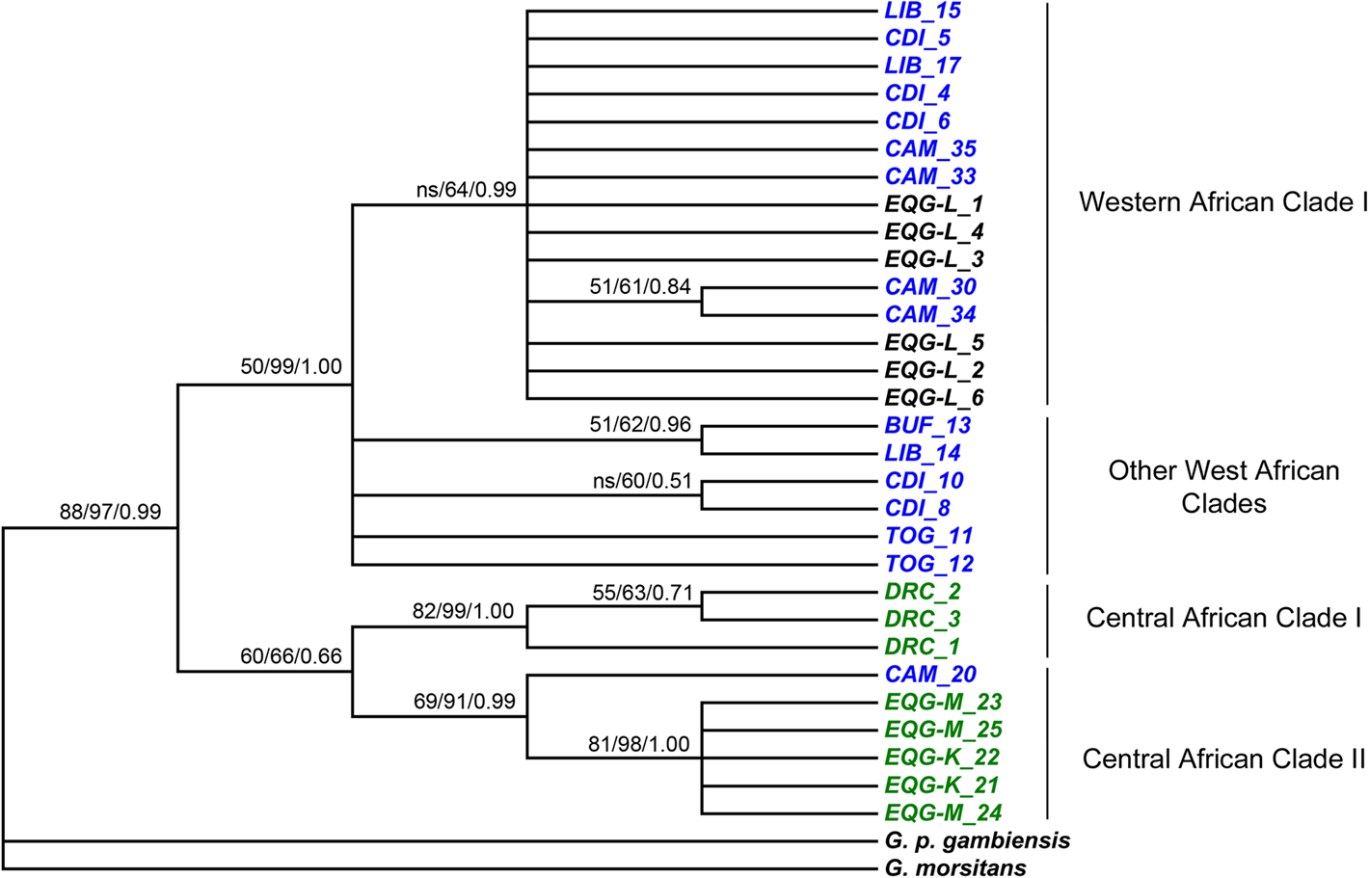
**Phylogenetic reconstruction inferred for the *G. p. palpalis *COI dataset.** Majority rule of the maximum likelihood consensus, distance consensus and Bayesian trees inferred for the COI dataset. Models of evolution used are detailed in Table 1. Numbers shown next to the nodes are the support values, i.e. the percentage of bootstrap replications (500 replicates in maximum likelihood and 2000 in distance based analysis) and posterior probability in Bayesian trees. Analysis involved 622 positions in the final dataset. *EQG-L*: Equatorial Guinea, Luba focus, *EQG-M*: Equatorial Guinea, Mbini focus, *EQG-K*: Equatorial Guinea, Kogo focus, *DRC*: Democratic Republic of Congo, *CAM*: Cameroon, *LIB*: Liberia, *CDI*: Cote d’Ivoire, *TOG*: Togo, *BUF*: Burkina Faso. ns: no support (<50 bootstrap or 0.5 posterior probability).

ML, distance based NJ and BI trees showed common topologies. All mirrored two major geographic separated clusters from Central (CAC) and Western Africa (WAC) (Figure 2). NJ and BI trees showed more support in this split: 97% bootstrap and 0.9999 posterior probability in BI trees, respectively, than ML inference (88%). Within WAC, organisation was unclear and again NJ and BI methods yielded a more robust clustering. ML inference method failed to yield a clear discrimination between clades from Western Africa (50% bootstrap) whereas NJ and BI trees split samples from this area in different sister sympatric groups (with 99% bootstrap and 1.0000 posterior probability). Clusters obtained by the NJ and BI methods within the WAC are probably not artifacts since both trees separate exactly the same specimens and in spite of the lower power of the ML tree, the same trend is visible.

**Figure 3:**
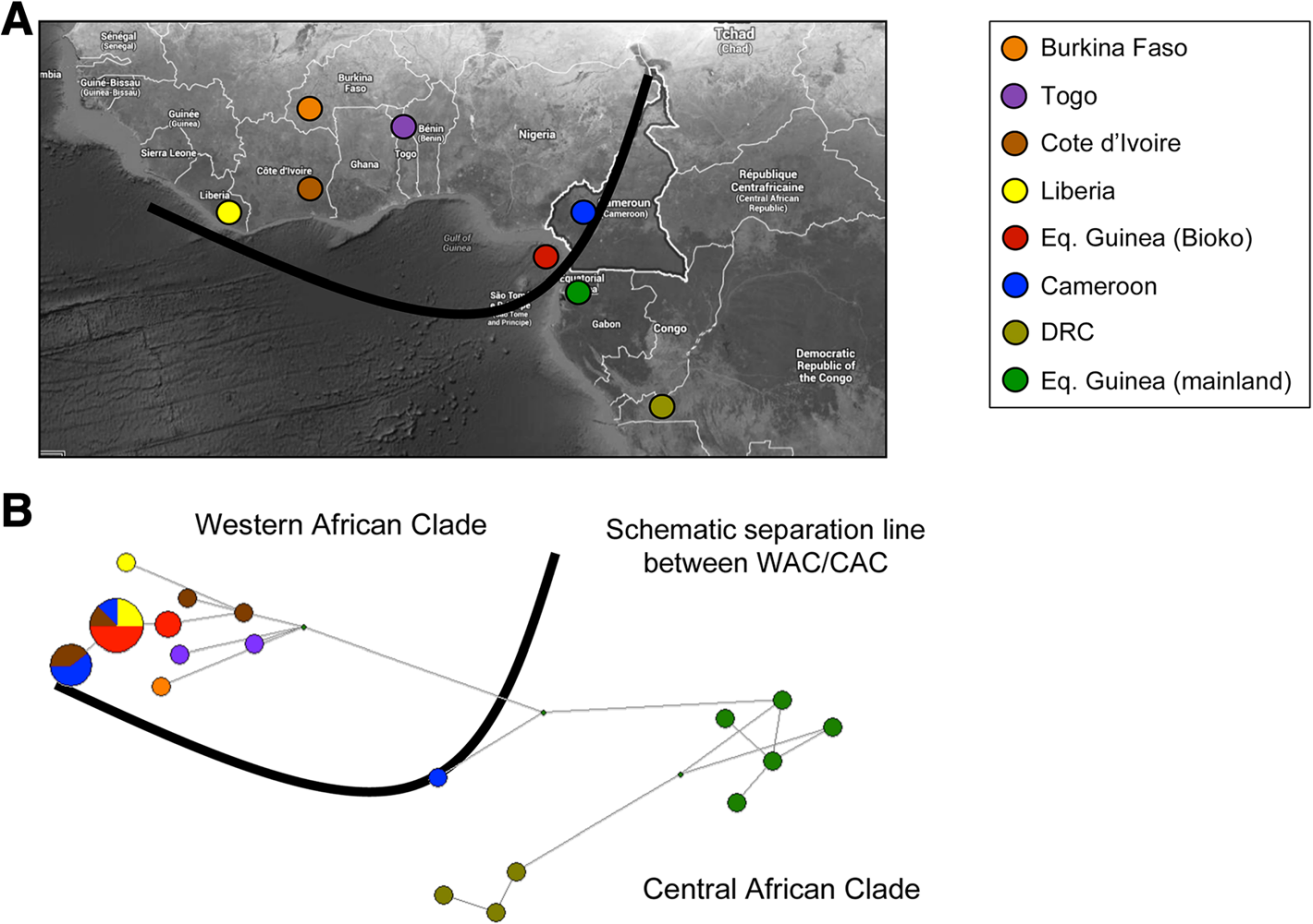
**Geographic representation of the COI haplotypes. A)** Geographic distribution of the COI lineages; **B)** Haplotypes network derived from 30 haplotypes of the *G. p. palpalis* complex. Haplotypes are represented by circles and their frequency is proportional to the area. Network diagrams created using Phylogenetic Network software from Fluxus and using the Median Joining method as described previously [47].

Regardless of the inference method used the COI haplotypes from the Luba focus grouped within the WAC I cluster in all trees, in clear contrast with the haplotypes from Kogo and Mbini foci (mainland Equatorial Guinea) that belong to the CAC cluster. In accordance to phylogeny trees, network analysis shows the clear separation of the two major geographic clusters (WAC and CAC) (Figure 3) together with the two subgroups corresponding to DRC and continental Equatorial Guinean haplotypes within the CAC (CAC I and II in Figure 2).

**Figure 4:**
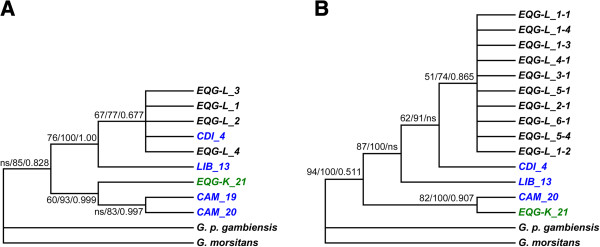
**Phylogenetic reconstruction inferred for the ND2 and concatenated COI + ND2 datasets.** Majority rule of the maximum likelihood consensus, distance consensus and Bayesian trees inferred for the **A)** ND2 and **B)**  concatenated COI + ND2 dataset. Models of evolution used are detailed in Table 1. Numbers shown next to the nodes are the support values, i.e. the percentage of bootstrap replications (500 replicates in maximum likelihood and 2000 in distance based analysis) and posterior probability in Bayesian trees. Analysis involved 501 (ND2) or 1123 (COI + ND2) positions in the final dataset. *EQG-L*: Equatorial Guinea, Luba focus, *EQG-K*: Equatorial Guinea, Kogo focus, *CAM*: Cameroon, *LIB*: Liberia, *CDI*: Cote d’Ivoire. ns: no support (<50 bootstrap or 0.5 posterior probability).

Regarding ND2 gene, five haplotypes were available in Genbank, one from Ivory Coast (EU591895), one from Liberia (EU591884), two from Cameroon (EU591897-8) and one from Equatorial Guinea (EU591905). In accordance with COI data, ML, distance based NJ and BI trees constructed with ND2 and COI + ND2 concatenated genes (Figure 4), exhibited a clear separation between the sequences of mainland Equatorial Guinean haplotypes and those from the Luba focus.

The 16S sequences were 100% identical in the 34 randomly selected individuals from Luba analysed by this marker. Only one *G. p. palpalis* 16S sequence, from Cameroon, was available in GenBank (EU591913) before this work. Alignment of the single 16S haplotype found in Luba was performed with this Cameroonian sequence and other *Glossina* taxa, namely *G. p. gambiensis* (EU591911.1), *G. fuscipes quanzensis* (EU591910.1), *G. fuscipes fuscipes* (EU591906.1), *G. tachinoides* (EU591917.1), *G. pallicera* (EU591918.1), *G. morsitans morsitans* (EU591920.1) and *G. pallidipes* (EU591925.1) (Additional file 3: Figure S1). This alignment revealed 1.91% distance between *G. p. palpalis* from Luba and Cameroon, and distances ranging between 2.4% (*G. p. gambiensis*) and 8.97% (*G. pallidipes*) when comparing Luba 16S haplotype with other *Glossina* taxa (Maximum Likelihood Composite Model [48]).

### Nuclear ITS1 marker

ITS1 size polymorphism was assessed for 59 flies. All PCR products analysed exhibited a unique size around 240 bp, similar to the size reported by Dyer *et al*. [11] when this fragment was amplified from flies belonging to the WAC. After sequencing, two similar genotypes defined by the (AT)_n_ microsatellite were found. Genotype 1 was characterized by (AT)_9_G(TA)_2_ region, whereas genotype 2 exhibited (AT)_10_ pure repeats (Additional file 4: Figure S2). As shown in Table 3, genotype 1 was dominant in all the 5 sampled areas of the Luba focus.

The two genotypes found were aligned with ITS1 sequences of *G. p. palpalis* from mainland Equatorial Guinea (Kogo: J767886 and Mbini: J767887-J767888), DRC (J767892 and J767893), Guinea Conakry (EU591930), Gambia (EU591931), Burkina Faso (EU591932), Togo (EU591933), Liberia (EU591934) and Ivory Coast (EU5991935) (Additional file 4: Figure S2). The main genotype, found in 44/59 flies and defined by a (AT)_9_G(TA)_2_ domain, was not detected in the other published sequences. The second genotype, defined by a (AT)_10_ repeat, shared 100% homology with the sequence of a Togo sample.

## Discussion

Luba focus is located at the edge between the two main clades of *G. p. palpalis*, one named WAC (for Western Africa Clade), including flies from Cameroon, Burkina Faso, Ivory Coast, Liberia and Togo; and the other, named CAC (for Central African Clade) represented by flies from the Democratic Republic of Congo and the mainland region of Equatorial Guinea. Phylogenetic analysis, using mtDNA markers, allowed us to cluster the *G. p. palpalis* population from Luba within the WAC. All the samples of this work were unambiguously included in this group and separated from the CAC regardless the phylogenetic inference method used.

Our results are consistent with the geological history of Bioko, originated by volcanic eruptions in the lower Tertiary period, around 60 million years BP. Bioko is a part of an archipelago which pertains to a large volcanic fracture originating in the South of Lake Chad and extending to Mount Cameroon in the continent [49]. Although politically belonging to Equatorial Guinea, Bioko lies closer to the Cameroon coast (*ca.* 30 km) rather than to the rest of Equatorial Guinea territories (more than 200 km from mainland and 700 km from Annobon island). During geoclimatic events of Quaternary period, Bioko was linked to mainland given the lower sea level, presumably to Cameroonian coast because of its geological origin. At the end of the last glaciation (around 12,000 years BP), Bioko was isolated by flooding and separated from the continent [50]. It is probable that *G. p. palpalis* population of Bioko was isolated from Cameroon coast after that event, as suggested for other insect vectors such as *Anopheles melas* and *Simulium yahense*[51,52].

Both mtDNA and nuclear markers show a very low genetic intra-variability. MtDNA genes data polymorphism did not exceed 0.8% and ITS1 sequences only yielded two closely related genotypes. For ITS1, the more abundant one is apparently exclusive of Luba focus, suggesting a certain degree of isolation. This is in accordance with previous studies, which found that *G. p. gambiensis* populations separated from the continent only by 4–5 km of sea show clear evidences of complete isolation. These results are based on wing landmarks, DNA mitochondrial markers and microsatellite dataset [53,54]. Our data should be also completed with an exhaustive coalescent population genetics analysis to support or not the hypothesis of an on-going allopatric speciation in Luba.

Because of the presence of these two sympatric ITS1 genotypes in Bioko Island one could speculate about the past occurrence of at least two separated migration events, as suggested for *Anopheles gambiae* in Bioko [55]. The ITS1 tandem array sequences are expected to be quickly homogenized by concerted evolution in interbreeding populations, whereas differences are usually observed between non-interbreeding ones [56,57]. However, the low difference observed between our ITS1 genotypes is based in the AT repeats, where a slight heterogeneity can be expected since microsatellite sequence could be evolving faster than the homogenization process. This phenomenon has been previously described in the tandem repeated U2 snRNA gene of primates [58,59].

Other possible explanation for the existence of two distinct ITS1 genotypes is a more recent reintroduction of tsetse flies from continent. Bioko Island is located at 30 km West of the Cameroonian coast, the closest area of the continent. Estimated active dispersal of *Glossina* sp. in one day is no longer than 1.3 km (in 15–30 min/day) [60,61]. Additionally, these flies are usually unable to flight for long periods but rather in short bursts lasting between 1 and 2 minutes [62,63]. These observations make extremely unlikely a recent (posterior to the glaciations period) reproductive contact between Luba and other continental foci due to active dispersal. However, human-mediated transportation of flies may not be disregarded. This situation allowed the reinvasion of *G. p. palpalis* in Principe Island in 1956 despite its eradication in 1914 using mobile traps carried by workers [2]. The distance between this island (*ca*. 240 km) and mainland is much larger than that of Bioko. It is therefore expected that human movement between Bioko and the coast of Cameroon has been much more frequent since the island was first colonized by the Bubi ethnical group, at the end of the last glaciation [64]. Finally, although the existence of ITS1 hybrid forms was initially suggested in *G. p. palpalis* of Equatorial Guinea [11], this hypothesis was later rejected by the same authors using microsatellite markers [10]. A set of highly polymorphic nuclear markers should be applied in the future to definitively test this hypothesis in Luba.

The genetic markers used to assess geographical structuring of *G. p. palpalis* demonstrate the existence of two allopatric taxa in Equatorial Guinea, one in the insular focus, Luba, and the other in two of the mainland foci (Kogo and Mbini). *G. p. palpalis* populations from Rio Campo, the third mainland focus of the country, have not been studied yet. In Equatorial Guinea, vector control was implemented in Kogo and Mbini from 2002 to 2009, whereas in Luba the active detection and treatment of HAT cases was the only control method employed [14]. Vector control has proved to be efficient at controlling HAT transmission [2,5,13] since tsetse flies show particular features that make them highly susceptible to direct interventions. Firstly, tsetse flies have a very low reproductive rate, given that a female individual has probably only one reproductive mating in its life and deposits only one larva per generation (up to 12 generations with 9–10 days of interval for a lifespan of 2–3 months) [2]. As a result of this low reproductive rate, the population of the vector is usually low comparing to other diptera and small increases in mortality can lead to control [4,65] or even to population extinction [66]. Secondly, the active dispersal of tsetse flies is generally low [60,61] resulting in a more difficult re-colonization of cleared areas. Thirdly, the genetic variability observed within populations tends to be reduced as well [67,68], probably as a consequence of both low reproductive rate and limited dispersal capacity, making more difficult for the selection of new attributes such as resistance to insecticides. There are, however, other behavioural features such as feeding preferences or trap-avoidance that can vary at subspecies and even at population level. For example, within *G. p. palpalis*, diverse feeding preferences across foci of Cameroon have been observed [69]. Although this observation could be attributed to the opportunistic feeding behaviour of *G. p. palpalis*[70], it could be also associated with the existence of genetically different *G. p. palpalis* populations, given the probable isolation of these foci [71]. Indeed, different feeding patterns in two sympatric populations of *G. p. palpalis* and *G. p. gambiensis* have also been described in preforest areas of Cote d’Ivoire [72], demonstrating that this feature can differ between closely related subspecies. The feeding behaviour of the vector can be crucial to design an effective vector control campaign since it provides valuable information to understand the epidemiological cycle of the parasite at local level.

## Conclusions

If cryptic species of *G. p. palpalis* are circulating as our data and previous observations suggest [11], the occurrence of both variants in Equatorial Guinea could have an important impact in the control of sleeping sickness. Are vector control methods equally effective for all foci? Do tsetse flies from Kogo and Mbini have different feeding preferences than those of Luba? Could Luba tsetse fly populations feed mainly in wild fauna allowing *T. b. gambiense* to persist in the focus even with absence of human infections? Are both groups equally competent for trypanosome transmission? An entomological study comparing feeding behaviour, habitat selection and parasite strain specificity of *G. p. palpalis* populations from the island and the continent should be conducted to ascertain if the genetic differences observed could be reflected on the vector ecology.

## Competing interests

Authors declare no competing interests.

## Authors’ contribution

CCO participated in the sample collection, molecular genetic studies, data analysis and wrote the manuscript. JK participated in statistical analysis. PN carried out the amplification of the genes and prepared the products for sequencing. NNB and PRNA participated in sample collection. MN, JC, PNA, JP, AB and JMB conceived the study, participated in its design and coordination, analysed the data and wrote the manuscript. All authors read and approved the final manuscript.

## Supplementary Material

Additional file 1: Table S1 Summary of primers used. Click here for file

Additional file 2: Table S2 Estimated genetic diversity, calculated in number of observed haplotypes. Click here for file

Additional file 3: Figure S1 Alignment of 16S found in *G. p. palpalis* from Luba focus. Gpp: *G. p. palpalis,* CAM: Cameroon, Gpg: *G. p. gambiensis*, Gfq: *G. fuscipes quanzensis*, Gff: *G. fuscipes fuscipes*, Gtac: *G. tachinoides*, Gpallic: *G. pallicera*, Gmm: *G. morsitans morsitans*, Gpallid: *G. pallidipes*. Click here for file

Additional file 4: Figure S2 Sequencing profiles and alignment of ITS1 genotypes found in Luba focus. Sequence profiles of ITS genotypes 1 and 2 (A and B, respectively). C) Alignment of ITS1 genotypes of *G. p. palpalis* from different origins. *EQG-L*: Equatorial Guinea, Luba focus, *EQG-M*: Equatorial Guinea, Mbini focus, *EQG-K*: Equatorial Guinea, Kogo focus, *DRC*: Democratic Republic of Congo, *LIB*: Liberia, *CDI*: Cote d’Ivoire, *TOG*: Togo, *GAM*: Gambia, *BUF*: Burkina Faso, *GUI*: Guinea. Click here for file
