# Supplementary material for: Glossina palpalis palpalis populations from Equatorial Guinea belong to distinct allopatric clades
Source: Parasit Vectors. 2014 Jan 17;7:31. doi: 10.1186/1756-3305-7-31 (PMC3898820; doi:10.1186/1756-3305-7-31)
Supplement: Additional file 1: Table S1 — Summary of primers used. [file 1756-3305-7-31-S1.doc]

| **Marker** | **Primers sequence (5’-3’)** | **Length** | **Reference** |
| --- | --- | --- | --- |
| **COI** | TTGATTTTTTGGTCATCCAGAAGT TGAAGCTTAAATTCATTGCACTAATC | 622 | Simon et al.1994;  Dyer et al. 2008 |
| **ND2** | TTAGCACCCTTAATATTAATC AAAAAATCGAACAGATTATAAT | 501 | This work |
| **16S** | GGTCCCTTACGAATTTGAATATATCCT ACATGATCTGAGTTCAAACCGG | 213 | Simon et al. 1994; Dyer et al. 2008 |
| **ITS1** | TGGACTTCGGATTAAGTACAACA TCATTATGCGCTATTAAGGTAAGC | 230-240 | Dyer et al. 2008 |

Table S1. Summary of primers used.
