# Supplementary material for: Glossina palpalis palpalis populations from Equatorial Guinea belong to distinct allopatric clades
Source: Parasit Vectors. 2014 Jan 17;7:31. doi: 10.1186/1756-3305-7-31 (PMC3898820; doi:10.1186/1756-3305-7-31)
Supplement: Additional file 2: Table S2 — Estimated genetic diversity, calculated in number of observed haplotypes. [file 1756-3305-7-31-S2.doc]

|  |  |  | Number of estimated haplotypes (standard error) | |
| --- | --- | --- | --- | --- |
| Areas | Number of flies | Number of observed haplotypes | jack1 (SE) | boot (SE) |
| LasPalmas | 10 | 5 | 8.6 (1.8) | 6.39 (0.86) |
| Fortuny | 10 | 5 | 8.6 (1.8) | 6.4 (0.86) |
| Fortuny Boloco | 10 | 3 | 4.8 (1.27) | 3.7 (0.65) |
| B. Drumen | 18 | 5 | 7.83 (1.64) | 6.19 (0.85) |
| B. Avendaño | 12 | 5 | 6.83 (1.3) | 6.04 (0.78) |
| Total | 60 | 10 | 13.93 (1.97) | 11.86 (1.09) |

Table S2. Estimated genetic diversity, calculated in number of observed haplotypes.
