# Supplementary figures and images for: Glossina palpalis palpalis populations from Equatorial Guinea belong to distinct allopatric clades
Source: Parasit Vectors. 2014 Jan 17;7:31. doi: 10.1186/1756-3305-7-31 (PMC3898820; doi:10.1186/1756-3305-7-31)

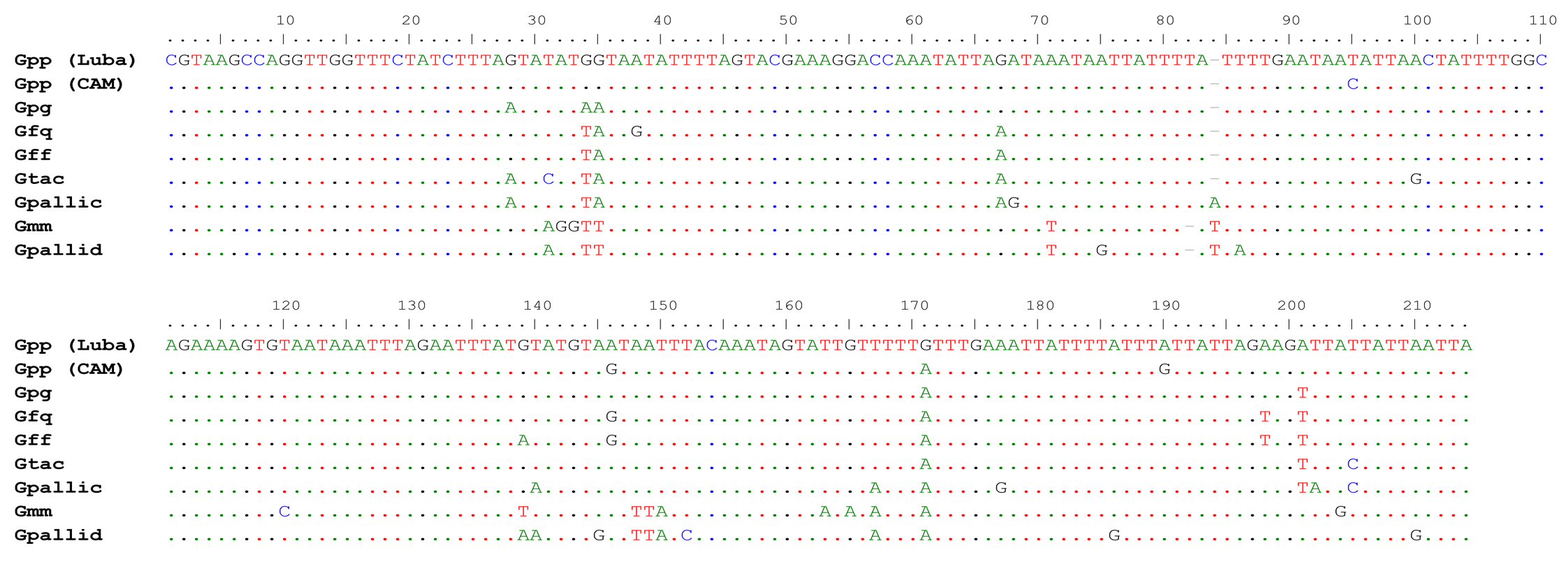

Supplement: Additional file 3: Figure S1 — Alignment of 16S found in G. p. palpalis from Luba focus. Gpp: G. p. palpalis, CAM: Cameroon, Gpg: G. p. gambiensis, Gfq: G. fuscipes quanzensis, Gff: G. fuscipes fuscipes, Gtac: G. tachinoides, Gpallic: G. pallicera, Gmm: G. morsitans morsitans, Gpallid: G. pallidipes. [file 1756-3305-7-31-S3.tiff]

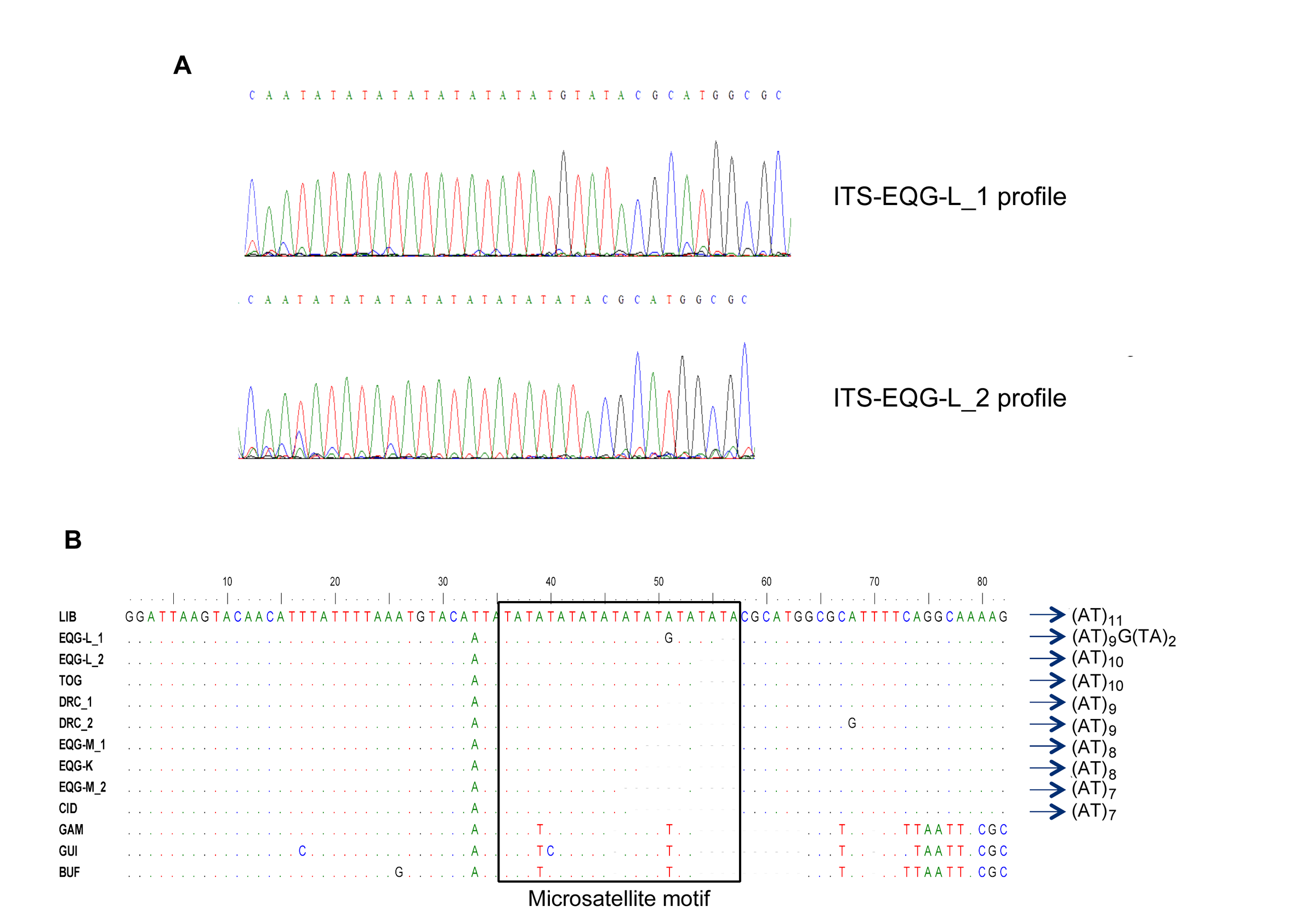

Supplement: Additional file 4: Figure S2 — Sequencing profiles and alignment of ITS1 genotypes found in Luba focus. Sequence profiles of ITS genotypes 1 and 2 (A and B, respectively). C) Alignment of ITS1 genotypes of G. p. palpalis from different origins. EQG-L: Equatorial Guinea, Luba focus, EQG-M: Equatorial Guinea, Mbini focus, EQG-K: Equatorial Guinea, Kogo focus, DRC: Democratic Republic of Congo, LIB: Liberia, CDI: Cote d’Ivoire, TOG: Togo, GAM: Gambia, BUF: Burkina Faso, GUI: Guinea. [file 1756-3305-7-31-S4.tiff]
